# Occupational therapy improves functional recovery and reduces delirium in critically ill adults with and without stroke: a systematic review and meta-analysis

**DOI:** 10.3389/fmed.2025.1733103

**Published:** 2026-02-19

**Authors:** Shuting Hua, Kunpeng Qiu, Shumin Zeng, Hui Wang, Tong Liu

**Affiliations:** 1 The Fifth Clinical College of Guangzhou University of Chinese Medicine, Guangzhou, China; 2 Guangdong Provincial Engineering Technology Research Institute of Traditional Chinese Medicine, Guangzhou, China; 3 Guangdong Provincial Second Hospital of Traditional Chinese Medicine, Guangzhou, China; 4 Department of Acupuncture and Rehabilitation, Guangdong Provincial Second Hospital of Traditional Chinese Medicine, Guangzhou, China; 5 Guangdong Provincial Key Laboratory of Research and Development in Traditional Chinese Medicine, Guangzhou, China

**Keywords:** critical care, ICU rehabilitation, post-intensive care syndrome, occupational therapy, activities of daily living, delirium, randomized controlled trials, systematic review and meta-analysis

## Abstract

**Background:**

Post-intensive care syndrome (PICS), encompassing physical, psychological, and cognitive impairments, significantly compromises recovery in critical illness survivors. Although occupational therapy (OT) may mitigate functional decline, its efficacy in the intensive care unit (ICU) remains inadequately established. This systematic review and meta-analysis evaluated the impact of OT on clinical outcomes in critically ill adults.

**Methods:**

We searched PubMed, Embase, Web of Science, CINAHL, Cochrane Library, CNKI, Wanfang, and China Biomedical Literature Service System from inception to August 5, 2025 for randomized controlled trials (RCTs) assessing ICU-based OT. Primary outcomes included activities of daily living (ADL), delirium incidence, grip strength, and mechanical ventilation duration. Data were synthesized using RevMan 5.3, with continuous outcomes expressed as mean difference (MD) or standardized mean difference (SMD) and dichotomous outcomes as relative risk (RR), all with 95% confidence intervals (CIs). The certainty of evidence for each outcome was assessed using the GRADE approach.

**Results:**

Pooled analyses demonstrated that OT significantly enhanced ADL performance [SMD = 0.72, 95% CI (0.40, 1.05), *p* < 0.001], reduced the incidence of delirium [RR = 0.44, 95% CI (0.30, 0.63), *p* < 0.001], increased grip strength [MD = 3.90 kg, 95% CI (2.03, 5.76), *p* < 0.001], and shortened the duration of mechanical ventilation [SMD = −0.68, 95% CI (−0.99, −0.37), *p* < 0.001]. The certainty of evidence (GRADE) was low for ADL, and moderate for delirium, grip strength, and mechanical ventilation duration. Subgroup analysis of stroke patients further demonstrated that OT significantly improved ADL performance [SMD = 0.81, 95% CI (0.42, 1.20), *p* < 0.001] and reduced delirium incidence [RR = 0.39, 95% CI (0.21, 0.72), *p* = 0.003], suggesting a particularly beneficial effect in this vulnerable population.

**Conclusion:**

This meta-analysis provides evidence that OT may enhance functional recovery, prevent delirium, and facilitate weaning in critically ill patients. However, the strength of the evidence is low to moderate, tempered by the limited number of trials, risk of bias, and observed heterogeneity. These findings underscore the need for further rigorous investigation to establish optimal protocols.

**Systematic review registration:**

https://www.crd.york.ac.uk/PROSPERO/recorddashboard, Unique identifier: CRD42025312345.

## 1 Introduction

Admission to the intensive care unit (ICU) exposes patients to profound physiological and psychological stressors, including a confined environment, immobility, pain, and sedative exposure. These factors collectively predispose individuals to develop persistent deficits across multiple domains, a condition recognized as post-intensive care syndrome (PICS) (1). PICS is strongly associated with adverse long-term outcomes, including ICU-acquired weakness (ICU-AW), delirium, cognitive impairment, post-traumatic stress disorder (PTSD), increased rehospitalization and mortality, and diminished quality of life (2). Epidemiological studies indicate that approximately 64% of ICU survivors experience at least one functional impairment 3 months post-discharge (3), with 44–58% reporting significant deficits in basic self-care persisting for up to 12 months, thereby impeding a return to independence and imposing substantial burdens on families and healthcare systems (4). Consequently, developing effective, evidence-based strategies to prevent and mitigate PICS represents a critical priority in critical care medicine.

The evolution of healthcare models toward a biopsychosocial framework has intensified the focus on early, proactive rehabilitation. Concurrently, biomedical advances provide a stronger scientific basis for optimizing rehabilitation strategies (5), reinforcing the rationale for early, multidisciplinary interventions like occupational therapy (OT). OT is a client-centered health profession focused on promoting health through engagement in purposeful activities (6). To restore physical function, occupational therapists implement graded, task-oriented training tailored to patient capacity, progressing from fine motor tasks to complex activities, thereby countering functional decline from ICU-AW. For cognitive dysfunction, therapists employ targeted interventions like memory games and orientation exercises to enhance attention, memory, and executive function. Psychologically, engagement in meaningful occupations can restore autonomy and reduce anxiety. In group settings, OT can foster social readjustment and potentially lower PTSD risk. Ultimately, OT aims to improve functional capacity for self-care, work, and community reintegration through sensory, motor, and cognitive training, and environmental modifications (7, 8). Distinct from physical therapy’s focus on gross motor function, OT emphasizes holistic enhancement of quality of life and complex social function. A comprehensive synthesis of evidence regarding OT’s effect on the multifaceted prognosis of critically ill patients was previously lacking.

While prior reviews have explored critical care rehabilitation broadly, few have specifically synthesized evidence for OT’s effect on multidimensional outcomes. A recent meta-analysis (8) focused on delirium but found no significant effect of OT, a discrepancy potentially due to methodological differences. To address this gap, this review provides an updated, exhaustive synthesis, incorporating a broader range of international databases and rigorously assessing OT’s aggregate impact on functional, cognitive, and physiological outcomes.

Thus, this systematic review and meta-analysis was conducted to rigorously evaluate OT’s efficacy in improving health outcomes in critically ill ICU patients, providing a robust evidence base to inform clinical practice and policy.

## 2 Methods

This review was planned and executed in strict accordance with the Preferred Reporting Items for Systematic Reviews and Meta-Analyses (PRISMA) 2020 guidelines (9). The study protocol was registered *a priori* with the PROSPERO international prospective register of systematic reviews (Registration ID: CRD420251170109). As all analyzed data were extracted exclusively from previously published, ethically approved studies, separate ethical approval for this synthesis was not required.

### 2.1 Inclusion and exclusion criteria

The study selection criteria were explicitly defined using the well-established PICO (Population, Intervention, Comparison, Outcome) framework:

Population (P): Critically ill patients aged ≥18 years who were formally admitted to an ICU for a period exceeding 24 h.

Intervention (I): Occupational therapy, delivered either as a standalone therapeutic regimen or as a core, clearly definable component within a broader multidisciplinary intervention protocol where the distinct OT contribution could be accurately described.

Comparison (C): Routine ICU care, which could encompass standard physical therapy and/or nursing care as directed by the primary medical team, but explicitly excluded the specific, protocolized OT intervention under investigation.

Outcomes (O): The pre-specified primary outcome was the level of independence in activities of daily living (ADL), measured using validated instruments such as the Barthel Index or the Functional Independence Measure (FIM). Secondary outcomes of interest included the incidence of delirium (assessed using the Confusion Assessment Method for the ICU [CAM-ICU]), grip strength (objectively measured in kilograms using a standardized hand dynamometer), and the total duration of mechanical ventilation (recorded in hours or days).

Study Design: Only randomized controlled trials (RCTs) were considered for inclusion to maximize internal validity. Exclusion criteria comprised: duplicate publications, non-Chinese/English language publications, unavailable full-text articles, conference abstracts, dissertations, case reports, and non-randomized observational studies.

### 2.2 Search strategy

A comprehensive and systematic search strategy was developed in collaboration with an experienced academic librarian. Two reviewers independently performed systematic electronic searches in the following databases from their inception to August 5, 2025: PubMed, Embase, Web of Science, CINAHL, Cochrane Central Register of Controlled Trials, CNKI, Wanfang Database, and SinoMed. The search strategy strategically combined relevant controlled vocabulary terms [e.g., Medical Subject Headings (MeSH) in PubMed, Emtree in Embase] with extensive free-text keywords related to three core concepts: (1) intensive/critical care, (2) occupational therapy, and (3) randomized controlled trials. No restrictions were applied concerning publication date or status. The complete, detailed search strategy utilized for PubMed is provided in Supplementary Table 1 and was logically adapted for syntax and terminology across all other databases.

### 2.3 Study selection and data extraction

Two reviewers, both formally trained in evidence-based practice and systematic review methodology, independently screened all retrieved records for potential eligibility. Following the removal of duplicate citations using EndNote 20 software, the titles and abstracts of all remaining records were screened for preliminary relevance. The full-text articles of all potentially eligible studies were then procured and thoroughly assessed against the pre-specified inclusion and exclusion criteria. Any disagreements arising at any stage of the selection process were resolved through iterative discussion or, when necessary, by consulting a third senior reviewer. A standardized, piloted data extraction form was employed to systematically collect the following information from each included study: (1) basic study characteristics (title, first author, country, year of publication, trial registration number); (2) detailed participant information (total sample size, specific ICU type, patient demographics, baseline illness severity scores); (3) comprehensive intervention characteristics (precise description of both the OT and control interventions, including timing of initiation, session frequency, total intervention duration, and provider credentials); and (4) complete outcome data (means, standard deviations, and event counts for all relevant outcomes at all reported assessment time points). Where data were incomplete or unclear, corresponding authors were contacted via email on two separate occasions to request the missing information. Five studies were excluded due to unavailable data despite our attempts to contact the authors.

### 2.4 Risk of bias assessment

The methodological quality and risk of bias of each included RCT were independently evaluated by two reviewers using the Cochrane Risk of Bias tool for randomized trials (RoB 2.0) (10). This rigorous tool assesses potential bias across five key domains: bias arising from the randomization process, bias due to deviations from the intended interventions, bias due to missing outcome data, bias in the measurement of the outcome, and bias in the selection of the reported result. For each study, explicit judgments for each domain were made, culminating in an overall risk of bias judgment, categorized as ‘low risk of bias’, ‘some concerns’, or ‘high risk of bias’. Any disagreements between reviewers were resolved through consensus to reach a final rating.

### 2.5 Data synthesis and analysis

Meta-analysis was performed utilizing Review Manager (RevMan) software, version 5.3. For continuous outcomes, the mean difference (MD) was calculated when the same measurement instrument was used consistently across studies; conversely, the standardized mean difference (SMD) was employed when different, though conceptually similar, measurement tools were used, with conventional interpretations of SMD values of 0.2, 0.5, and 0.8 representing small, medium, and large effect sizes, respectively. For dichotomous outcomes, the relative risk (RR) was calculated as the measure of association. All pooled effect estimates are reported with their corresponding 95% confidence intervals (CIs). Statistical heterogeneity among the included studies was quantitatively assessed using the Chi^2^ test (employing a conservative significance level of *p* < 0.10 to indicate potential heterogeneity) and the I^2^ statistic, which quantifies the proportion of total variation attributable to heterogeneity. An I^2^ value of less than 50% was considered to indicate low heterogeneity, in which case a fixed-effect model was applied for pooling. An I^2^ value of 50% or higher was interpreted as indicating substantial heterogeneity, warranting the use of a more conservative random-effects model (utilizing the DerSimonian and Laird method) and prompting the performance of pre-specified subgroup analyses to explore potential clinical or methodological sources of heterogeneity [e.g., type of OT intervention (standalone vs. combined within multidisciplinary care), nature of the control group comparison, specific assessment tool used]. Sensitivity analyses were conducted by sequentially removing each individual study from the meta-analysis to assess the robustness and stability of the pooled results. A formal assessment of publication bias using funnel plots and statistical tests such as Egger’s test was originally planned but was subsequently deemed methodologically inappropriate due to the small number of studies (<10) included in each separate meta-analysis, as the statistical power of such tests is unacceptably low to reliably distinguish chance from real asymmetry under these conditions.

We also conducted a pre-specified subgroup analysis to evaluate the efficacy of OT in critically ill patients with stroke versus those without stroke, where data were available. This subgroup analysis aimed to explore whether the benefits of OT were consistent across these clinically distinct populations.

## 3 Results

### 3.1 Study selection

**Figure 1:**
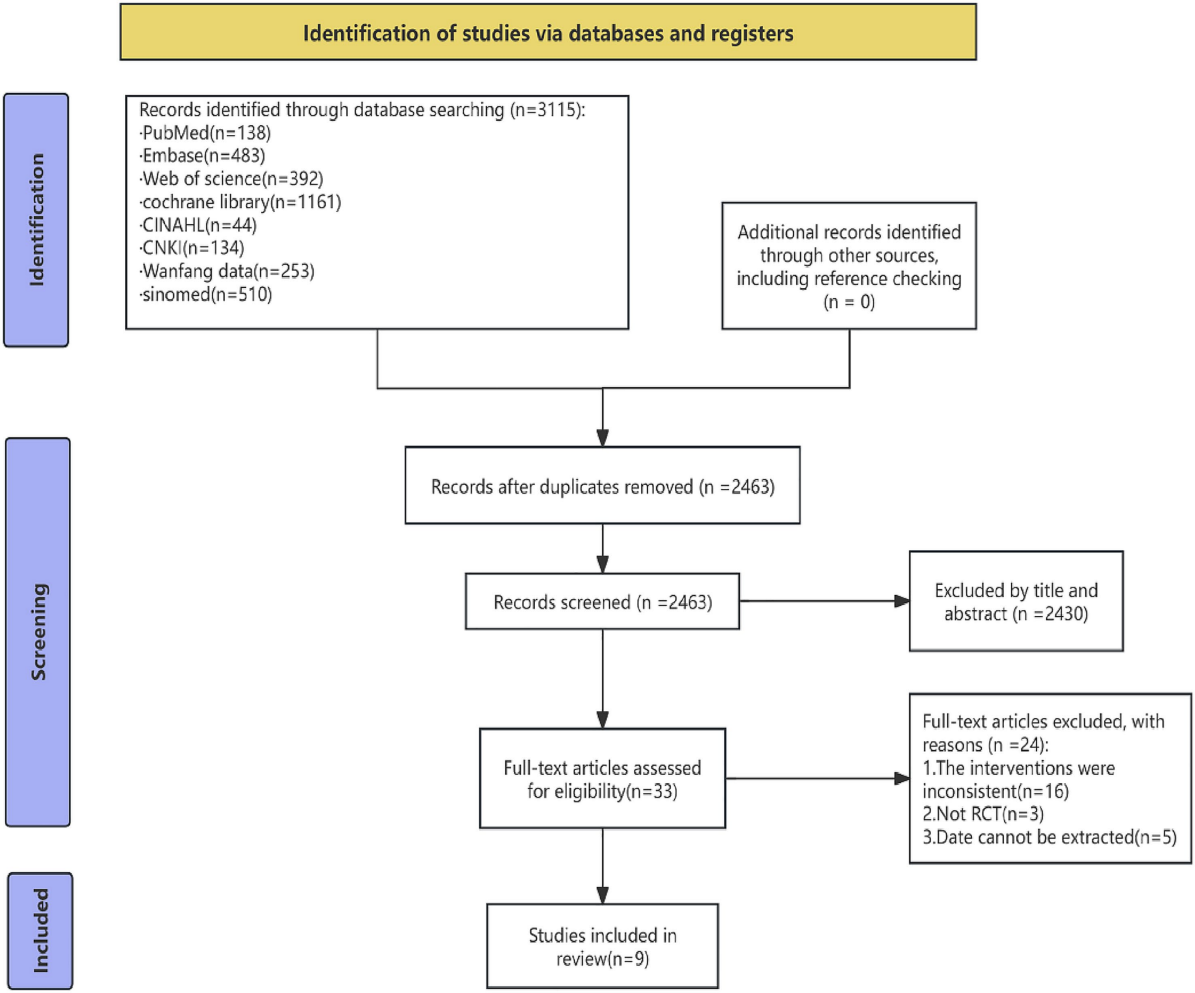
PRISMA flow diagram of study identification, screening, and inclusion. The diagram illustrates the process of study selection for the systematic review and meta-analysis, following the Preferred Reporting Items for Systematic Reviews and Meta-Analyses (PRISMA) 2020 guidelines. A total of 3,115 records were identified through systematic searches of electronic databases (PubMed, *n* = 138; Embase, *n* = 483; Web of Science, *n* = 392; Cochrane Library, *n* = 1,161; CINAHL, *n* = 44; CNKI, *n* = 134; Wanfang Data, *n* = 253; and SinoMed, *n* = 510). After the removal of 652 duplicates, 2,463 records were screened based on titles and abstracts. Subsequently, 33 full-text articles were assessed for eligibility. Of these, 24 studies were excluded with reasons (interventions inconsistent with the review’s definition of occupational therapy, *n* = 16; study design not an RCT, *n* = 3; essential data could not be extracted, *n* = 5). The final analysis included nine randomized controlled trials.

The initial execution of the systematic search strategy across all designated electronic databases yielded a total of 3,115 unique records. Following the removal of 652 duplicate citations, 2,463 records underwent preliminary screening based on their titles and abstracts. From this initial screening, 33 full-text articles were deemed potentially eligible and were retrieved for a detailed, in-depth eligibility assessment. Of these, 24 studies were excluded for the following reasons: interventions were inconsistent with the review’s definition of OT (*n* = 16), study design was not an RCT (*n* = 3), and essential data could not be extracted (*n* = 5). This process culminated in the final inclusion of nine RCTs that fully met all inclusion criteria, encompassing a total study population of 819 critically ill patients. The complete, sequential study selection process is delineated in the PRISMA flow diagram (Figure 1).

### 3.2 Characteristics of included studies

The key methodological and clinical characteristics of the nine included RCTs are comprehensively summarized in Table 1. Collectively, the studies randomized 409 patients to experimental (OT) groups and 410 patients to control groups. The mean age of participants, where reported, was predominantly over 50 years across the included study cohorts. All described interventions were delivered within a hospital-based ICU setting. The control groups uniformly received some form of routine care, which, depending on the specific ICU protocol, could include elements of physical therapy. The experimental interventions were categorized as consisting of OT alone in four trials (11–14) and OT integrated with physical therapy in five trials (15–19). The most frequently reported core component of the OT interventions was ADL training (11–15, 17–19) (implemented in 8 RCTs, *n* = 757 patients), typically involving practical, graded exercises such as dressing, grooming, beading, or manipulating buttons. Cognitive interventions (11–13) (employed in 3 RCTs, *n* = 249 patients), including structured activities like card games, multisensory stimulation, and memory retraining exercises, were also commonly incorporated. One trial (16) did not provide specific details regarding the constituent OT activities. Methodological quality indicators revealed that five of the included trials (11, 13, 15–17) had prospectively registered their study protocols, four trials (11, 13, 15, 17) explicitly reported conducting an *a priori* sample size calculation, and five trials (12, 13, 15–17) incorporated a designated follow-up period beyond the immediate ICU stay.

**Table 1:** Basic characteristics of included trials.

Author	Year	Country	Sample size (T/C)	Average age	ICU type	Subject type	Intervention	Outcomes
Schweickert et al. (15)	2009	America	49/55	54.16	ICU	Mechanically ventilated patients	Passive movement, bed movement, ADL training, bedside rotation training, walking training	Activities of daily living, delirium, grip strength, duration of mechanical ventilation
Alvarez et al. (11)	2017	Chile	65/65	68.88	ICU	Elderly non-intubated patients	Multisensory stimulation, cognitive stimulation, ADL training, limb function exercises, family involvement	Activities of daily living, delirium
Jiang and Tang (12)	2018	China	46/43	37.65	ICU	Neurosurgical patients	Psychological intervention, cognitive function exercise, ADL training	Activities of daily living
Wu et al. (16)	2019	Australia	29/33	53.9	ICU	Not reported	Occupational therapy provided by occupational therapist, but intervention content not reported	Not reported
Patel et al. (17)	2023	America	99/99	55.54	Surgical ICU	Mechanically ventilated patients	Passive mobility, bed mobility, ADL training. 25–30 min each time	Activities of daily living, duration of mechanical ventilation
Rapolthy-Beck et al. (13)	2023	Australia	15/15	59.6	ICU	Mechanically ventilated patients	ADL training, leisure activities, cognitive stimulation, sensory stimulation	Activities of daily living, duration of mechanical ventilation
Yu et al. (18)	2018	China	38/34	73.37	ICU	Mechanically ventilated patients	Early Mobility, Functional Training, ADL Training	Activities of daily living, grip strength, duration of mechanical ventilation
Tu (19)	2020	China	33/33	55.1	ICU	Cardiac surgery patients	Early Mobility, Functional Training, ADL Training	Delirium
Yu (19)	2019	China	35/33	76.48	ICU	Mechanically ventilated patients	Early Mobility, Functional Training, ADL Training	Delirium, duration of mechanical ventilation

### 3.3 Risk of bias assessment

**Figure 2:**
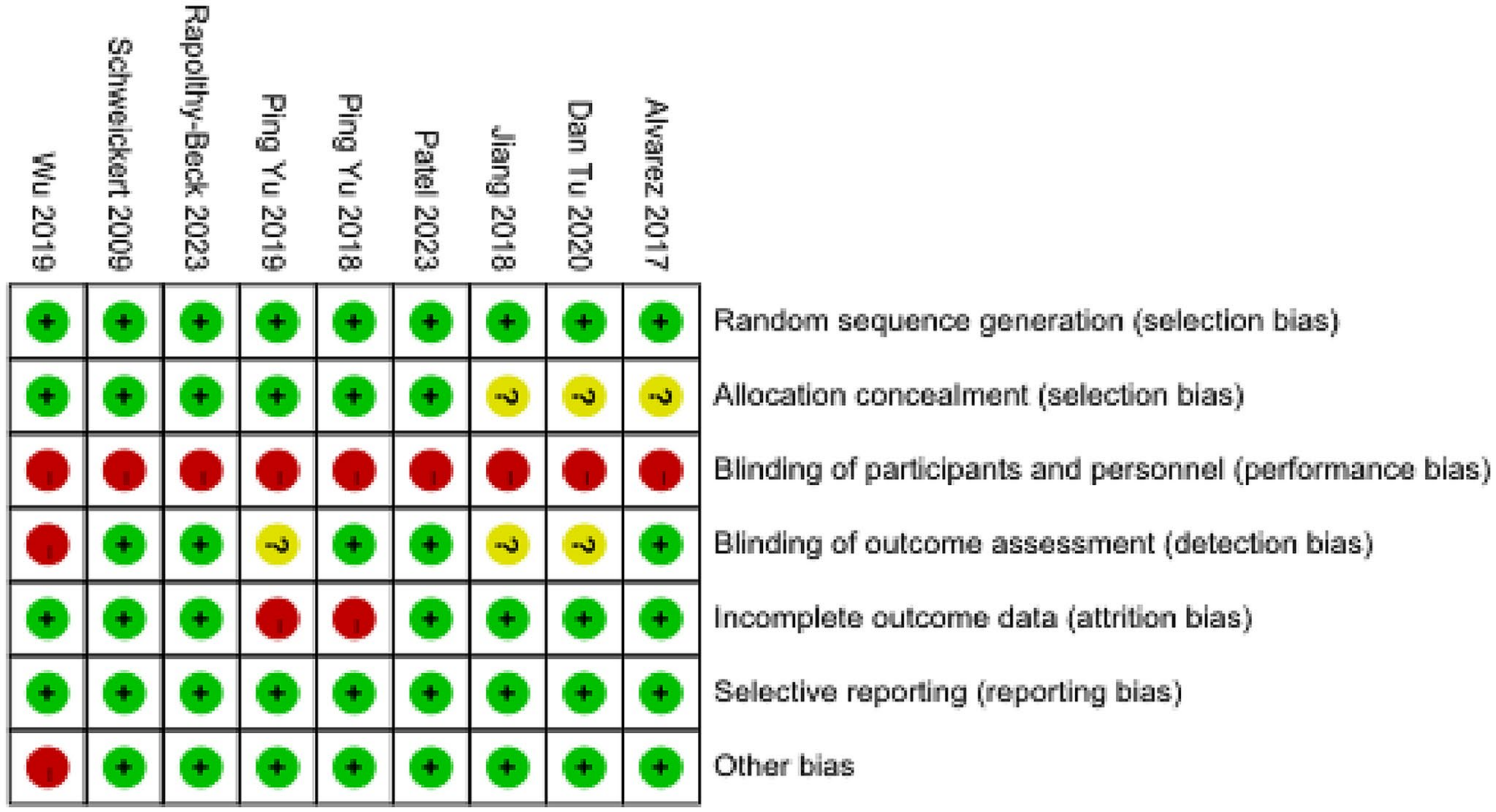
Risk of bias assessment for included randomized controlled trials. Risk of bias graph illustrating the distribution of judgments for each domain. The methodological quality of the nine included randomized controlled trials was independently assessed by two reviewers using the Cochrane Risk of Bias tool (RoB 2.0). Judgments for each domain are categorized as low risk (+), some concerns (?), or high risk (−). The overall risk of bias was judged as ‘high’ for all studies, primarily due to the inherent inability to blind participants and personnel delivering the complex occupational therapy intervention, leading to a high risk of bias in the ‘deviations from intended interventions’ domain.

The results of the methodological quality assessment using the Cochrane RoB 2.0 tool are presented graphically in Figure 2. Concerning the domain of bias arising from the randomization process, five trials (11, 13, 15–17) were judged to be at low risk, typically due to the reported use of computer-generated random sequence generation and adequate allocation concealment. The remaining four studies (12, 14, 18, 19) provided insufficient methodological detail regarding the randomization procedure and were consequently rated as having ‘some concerns’. Due to the inherent nature of the complex OT intervention, blinding of participating patients and intervening therapists was not pragmatically feasible in any of the included studies, leading to an unequivocal rating of ‘high risk’ for the domain of bias due to deviations from the intended interventions across all studies. The blinding of outcome assessors was explicitly reported and judged to be at low risk of bias in five trials (11, 13, 15, 17, 18) for objectively measured outcomes such as grip strength and ventilation duration; however, for more subjective, patient-reported outcomes like ADL, the risk of measurement bias was generally considered high. Incomplete outcome data were adequately addressed with appropriate statistical methods in seven studies (11–13, 15–17, 19), whereas two studies (14, 18) demonstrated notable imbalances in missing data between the intervention and control groups, raising some concerns regarding potential attrition bias. Selective reporting of results was deemed to be at low risk for all included studies, as the pre-specified primary outcomes outlined in the methods sections were consistently reported in the results. The overall risk of bias judgment was consequently ‘high’ for all nine studies, a determination primarily driven by the unavoidable lack of blinding for participants and personnel.

### 3.4 Results of meta-analyses

#### 3.4.1 Activity of daily living

**Figure 3:**
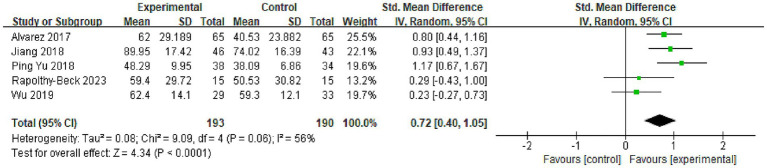
Forest plot for the effect of occupational therapy on activities of daily living. Meta-analysis of the effect of occupational therapy (OT) on Activities of Daily Living (ADL) compared to the control group. The effect size is expressed as Standardized Mean Difference (SMD) with a 95% confidence interval (CI). Data were pooled using a random-effects model due to substantial statistical heterogeneity among the five included studies (*I*^2^ = 56%, *p* = 0.06). The diamond represents the overall pooled effect estimate [SMD = 0.72, 95% CI (0.40, 1.05), *p* < 0.001], indicating a statistically significant, moderate-to-large improvement in ADL performance favoring the OT intervention. Experimental, experimental group; Control, control group; IV, Inverse Variance method.

Data from five trials provided evaluable data on activities of daily living for meta-analysis. The analysis indicated substantial statistical heterogeneity among these studies (*I*^2^ = 56%, *p* = 0.06), justifying the application of a random-effects model for pooling. The meta-analysis revealed a statistically significant and moderate-to-large positive effect favoring the OT intervention groups over the control groups [SMD = 0.72, 95% CI (0.40, 1.05), *p* < 0.001; Figure 3]. To explore the sources of heterogeneity, pre-specified subgroup analyses were conducted. These analyses suggested that the specific type of assessment tool utilized (Barthel Index versus FIM) was a potential modifier of the effect size and a contributor to heterogeneity, with the study by Wu et al. (12) identified as a notable outlier (Table 2). Unfortunately, a planned meta-regression to further investigate sources of heterogeneity (e.g., intervention intensity, duration, baseline severity) was not feasible due to the limited number of studies (*n* = 5) and inconsistent reporting of potential effect modifiers across the included trials. A sensitivity analysis, which involved iteratively removing each study from the model, confirmed that the overall finding of a statistically significant benefit remained robust, although the pooled point estimate exhibited some expected variation. It is noteworthy that two additional studies (15, 17) reported a significantly higher proportion of patients achieving functional independence in the OT group (*p* < 0.05) but could not be incorporated into the quantitative meta-analysis due to incompatible data presentation formats (e.g., reporting only medians and interquartile ranges), which qualitatively supports the meta-analysis findings.

**Table 2:** Summary table of activities of daily living subgroup analysis.

Subgroup	Category	Number of included trials	SMD(95%CI)	Heterogeneity
Measuring tools	FIM	3	0.50 (0.09,0.91)	*I*^2^ = 49% *p* = 0.14
	Barthel	2	1.03 (0.70,1.37)	*I*^2^ = 0% *p* = 0.49
Average age	Under 60 years old	3	0.52 (0.03,1.01)	*I*^2^ = 60% *p* = 0.08
	Over 60 years old	2	0.94 (0.59,1.29)	*I*^2^ = 27% *p* = 0.24

#### 3.4.2 Incidence of delirium

**Figure 4:**
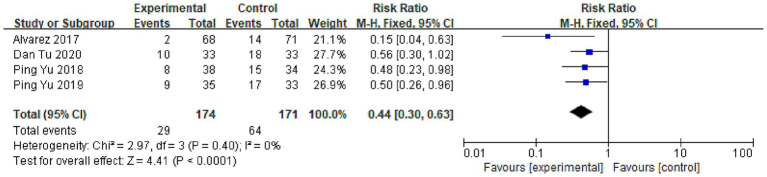
Meta-analysis of the effect of occupational therapy (OT) on the incidence of delirium compared to the control group. The effect size is expressed as Risk Ratio (RR) with a 95% confidence interval (CI). Data from four studies, all employing the Confusion Assessment Method for the ICU (CAM-ICU) for diagnosis, were pooled using a fixed-effect model (Mantel–Haenszel method) due to low statistical heterogeneity (*I*^2^ = 0%, *p* = 0.40). The pooled result demonstrates that OT was associated with a significant 56% reduction in the relative risk of delirium [RR = 0.44, 95% CI (0.30, 0.63), *p* < 0.001]. The diamond represents the overall pooled effect estimate.

Four trials assessed the incidence of delirium, uniformly employing the Confusion Assessment Method for the ICU (CAM-ICU) as the diagnostic instrument. Statistical heterogeneity among these studies was negligible (*I*^2^ = 0%, *p* = 0.40), permitting the use of a fixed-effect model for meta-analysis. The pooled results demonstrated that OT was associated with a pronounced 56% reduction in the relative risk of developing delirium during the ICU stay [RR = 0.44, 95% CI (0.30, 0.63), *p* < 0.001; Figure 4]. This finding remained robust and virtually unchanged in a subsequent sensitivity analysis, confirming the stability of the result.

#### 3.4.3 Grip strength

**Figure 5:**
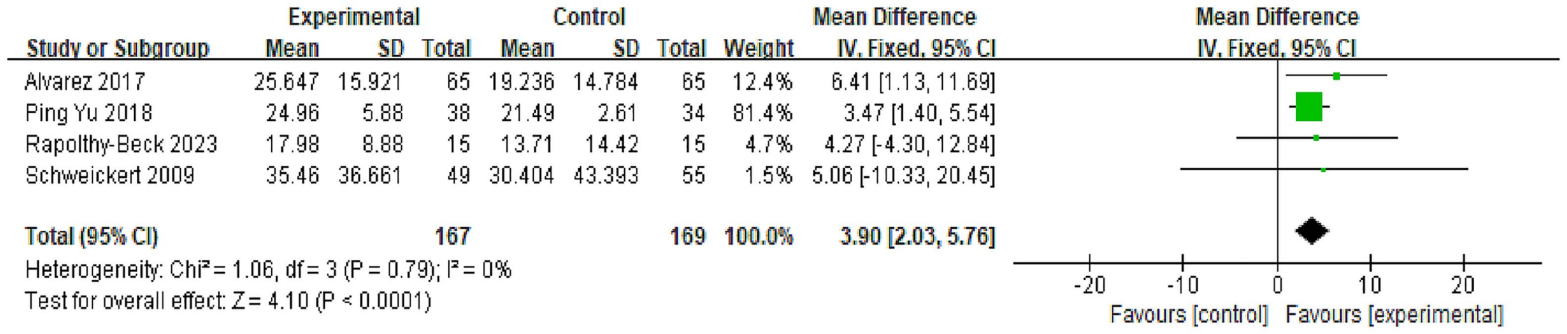
Meta-analysis of the effect of occupational therapy (OT) on grip strength compared to the control group. The effect size is expressed as Mean Difference (MD) in kilograms with a 95% confidence interval (CI), as all included studies used hand dynamometers for objective measurement. Data from four studies were pooled using a fixed-effect model (Inverse Variance method) due to low statistical heterogeneity (*I*^2^ = 0%, *p* = 0.79). The analysis revealed a statistically significant and clinically relevant improvement in grip strength favoring the OT groups [MD = 3.90 kg, 95% CI (2.03, 5.76), *p* < 0.001]. The diamond represents the overall pooled effect estimate.

Four trials provided quantitative data on grip strength, measured in kilograms using hand dynamometers. No appreciable statistical heterogeneity was detected among these studies (*I*^2^ = 0%, *p* = 0.79). The fixed-effect model meta-analysis revealed that OT interventions resulted in a statistically significant and clinically relevant improvement in grip strength, with a mean difference of 3.90 kilograms favoring the OT groups [MD = 3.90 kg, 95% CI (2.03, 5.76), *p* < 0.001; Figure 5].

#### 3.4.4 Duration of mechanical ventilation

**Figure 6:**
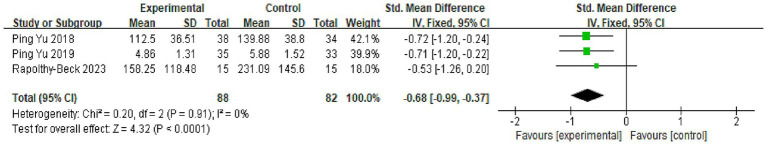
Forest plot for the effect of occupational therapy on the duration of mechanical ventilation. Meta-analysis of the effect of occupational therapy (OT) on the duration of mechanical ventilation compared to the control group. The effect size is expressed as Standardized Mean Difference (SMD) with a 95% confidence interval (CI) because the outcome was reported in varying units (hours or days) across studies. Data from three studies were pooled using a fixed-effect model (Inverse Variance method) due to low statistical heterogeneity (*I*^2^ = 0%, *p* = 0.91). The meta-analysis indicated that OT was associated with a statistically significant shortening of the mechanical ventilation period [SMD = −0.68, 95% CI (−0.99, −0.37), *p* < 0.001]. The diamond represents the overall pooled effect estimate.

Three trials reported data on the duration of mechanical ventilation suitable for meta-analysis. These studies exhibited low statistical heterogeneity (*I*^2^ = 0%, *p* = 0.91). The fixed-effect model meta-analysis indicated that OT was associated with a statistically significant shortening of the mechanical ventilation period, reflected by a moderate negative standardized mean difference [SMD = −0.68, 95% CI (−0.99, −0.37), *p* < 0.001; Figure 6]. A sensitivity analysis, sequentially removing each study, did not materially alter this conclusion. Two other studies (15, 17) reported ventilation duration using median and interquartile range, which precluded their inclusion in the primary meta-analysis; of these, one study (15) found a statistically significant reduction in ventilation time with OT (*p* = 0.02), while the other (17) reported a non-significant trend (*p* = 0.11).

#### 3.4.5 Subgroup analysis in stroke patients

**Figure 7:**
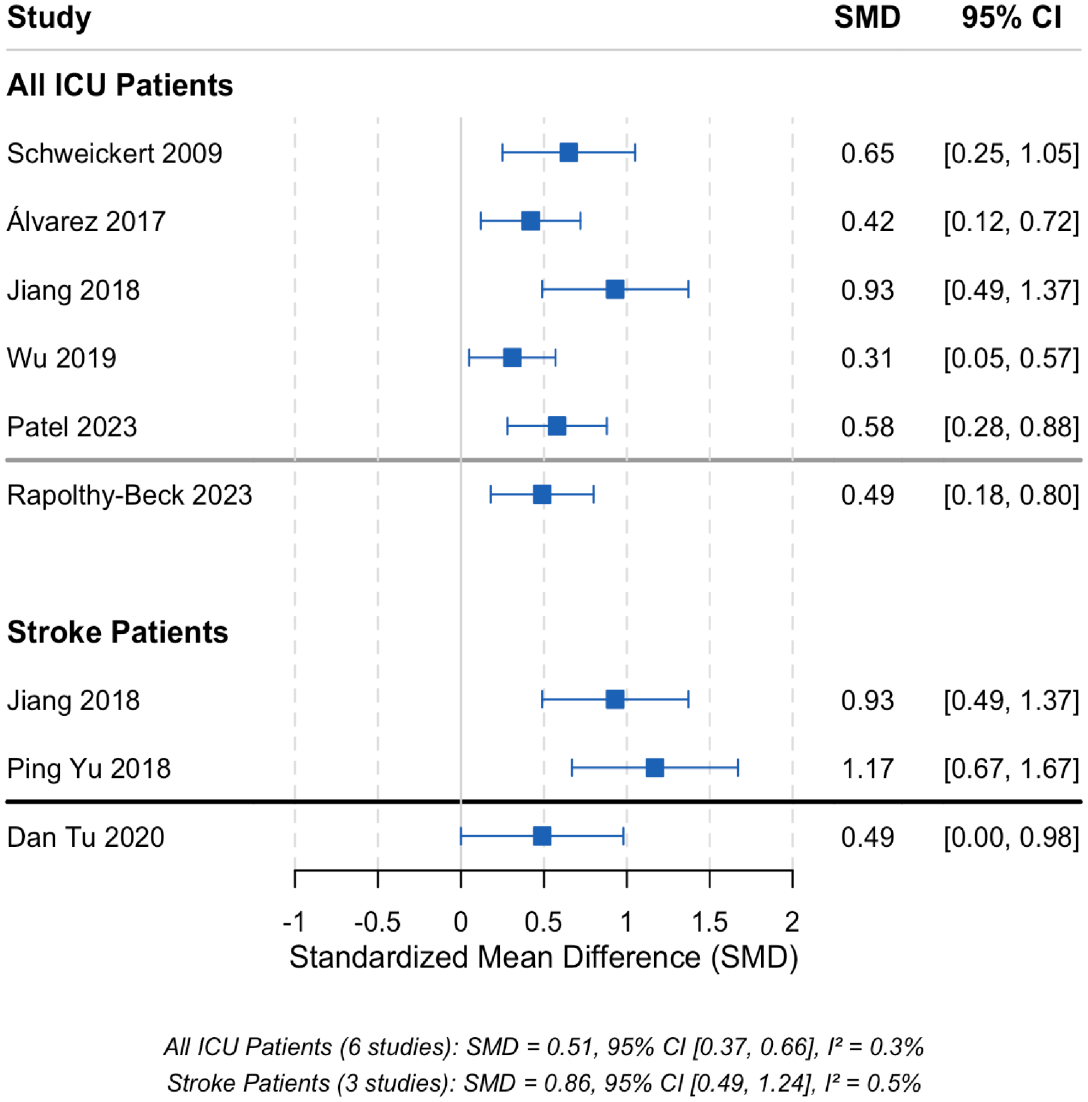
Subgroup analysis of the effect of occupational therapy on activities of daily living in critically ill adults: all ICU patients versus stroke patients. Forest plot shows standardized mean differences (SMD) with 95% confidence intervals (CIs). Blue squares and lines represent individual studies and pooled estimate for all ICU patients [6 studies; random-effects model, SMD = 0.72, 95% CI (0.40, 1.05), *I*^2^ = 56%]. Red squares and lines represent stroke patient subgroup [3 studies; random-effects model, SMD = 0.81, 95% CI [0.42, 1.20], *I*^2^ = 30%]. The vertical dashed line at SMD = 0 indicates no effect.

Among the included trials, three studies (12, 18, 19) enrolled patients with neurosurgical or stroke-related critical illness, allowing for a subgroup analysis. The results indicated that OT had a large and statistically significant effect on improving ADL in stroke patients [SMD = 0.81, 95% CI (0.42, 1.20), *p* < 0.001; Figure 7], which was greater than the overall effect observed in the mixed ICU population. Similarly, for delirium prevention, the protective effect of OT was particularly pronounced in stroke patients [RR = 0.39, 95% CI (0.21, 0.72), *p* = 0.003], suggesting that OT may be especially effective in this subgroup.

### 3.5 Sensitivity analysis and publication Bias

Sensitivity analyses, performed by systematically omitting one study at a time from each meta-analysis, confirmed that the pooled estimates for all primary outcomes remained statistically significant and stable in direction and magnitude, with the exception of the ADL outcome. For ADL, while statistical significance (*p* < 0.05) was consistently maintained, the precise point estimate of the SMD exhibited expected minor fluctuations, a common occurrence when substantial heterogeneity is present. This suggests that no single study was entirely responsible for the observed heterogeneity or the overall significant finding, though the result should be interpreted with an understanding of its underlying variability. As pre-specified in the protocol, a formal quantitative assessment of potential publication bias using funnel plots and associated statistical tests (e.g., Egger’s test) was not undertaken. This decision was based on the well-established methodological principle that such tests possess unacceptably low statistical power and are prone to misleading conclusions when the number of contributing studies in a meta-analysis is fewer than 10 (Supplementary Tables 2a,b).

### 3.6 Certainty of evidence

The overall certainty of evidence for each primary outcome was assessed using the GRADE framework and is summarized in the Summary of Findings table (Supplementary Table 3).

Activities of Daily Living (ADL): The evidence was rated as low certainty. It was downgraded by one level due to serious risk of bias (high risk of performance bias across all studies) and by one further level due to serious inconsistency (substantial unexplained statistical heterogeneity, *I*^2^ = 56%).

Incidence of Delirium: The evidence was rated as moderate certainty. It was downgraded by one level due to serious risk of bias (high risk of performance bias) but was not downgraded for other domains, as inconsistency, indirectness, and imprecision were not deemed serious.

Grip Strength: The evidence was rated as moderate certainty. It was downgraded by one level due to serious risk of bias (high risk of performance bias) but was not downgraded for other domains.

Duration of Mechanical Ventilation: The evidence was rated as moderate certainty. It was downgraded by one level due to serious risk of bias (high risk of performance bias) but was not downgraded for other domains.

Publication bias remained undetectable for all outcomes due to the limited number of included studies (<10).

## 4 Discussion

This systematic review and meta-analysis, synthesizing evidence from nine randomized controlled trials, provides compelling evidence that occupational therapy interventions are associated with statistically significant and clinically meaningful improvements across several pivotal health outcomes for critically ill patients recovering in the ICU. Specifically, our pooled analyses demonstrate that OT confers substantial benefits. However, the certainty of these findings varies, being moderate for delirium, grip strength, and mechanical ventilation duration, and low for ADL, indicating that the true effect for ADL might be substantially different.

The pronounced positive effect of OT on the recovery of ADL performance is highly consistent with the foundational principles and theoretical orientation of the occupational therapy profession, which explicitly focuses on enabling and empowering patients to (re)engage in meaningful, purposeful daily tasks and occupations. Our findings align coherently with the conclusions of prior systematic reviews by Zhao et al. (20) and Uceda-Portillo et al. (21), which also noted trends toward functional improvement. The recovery of independence in basic and instrumental ADL is of paramount importance, as persistent dependence in these fundamental activities is a well-established, powerful predictor of poor long-term patient outcomes, increased caregiver burden, and escalated healthcare costs (22). The substantial statistical heterogeneity observed in the ADL meta-analysis warrants careful consideration and interpretation. Our pre-specified subgroup analysis successfully identified the utilization of different ADL assessment tools—specifically the Barthel Index versus the Functional Independence Measure (FIM)—as a likely significant contributor to this observed variance. The Barthel Index primarily assesses basic self-care activities, whereas the FIM incorporates additional complex domains such as communication, social cognition, and community integration, thereby providing a broader, more comprehensive assessment of global disability. Furthermore, it is plausible that variations in the specific OT intervention protocols (e.g., standalone OT versus OT seamlessly integrated within a combined physical therapy regimen, differences in daily session duration, total intervention frequency, and overall program length) and divergent baseline patient characteristics across the included studies may have contributed to the underlying clinical and methodological heterogeneity. Despite our efforts to explore this through subgroup analysis, the residual heterogeneity highlights the challenge of synthesizing a cohesive estimate and suggests that the true effect of OT on ADL may vary depending on these contextual factors. The low certainty of evidence for this outcome underscores the need for cautious interpretation. Despite this observable methodological diversity, it is noteworthy that the direction of the treatment effect consistently favored the OT intervention across all analyzed subgroups, thereby reinforcing the fundamental robustness of the primary finding of benefit.

A particularly striking and potentially practice-informing finding from our analysis was the substantial reduction in the incidence of delirium associated with OT interventions. This robust result stands in clear contrast to the conclusions of a previous meta-analysis conducted by Zhao et al. (20), which reported no statistically significant effect of OT on delirium prevention. Several plausible factors may explain this notable discrepancy. First, our search strategy was deliberately more comprehensive and inclusive, incorporating systematic searches of major Chinese biomedical databases (CNKI, Wanfang, SinoMed) in addition to the standard international English-language databases, which may have captured a wider, more representative evidence base, including recent trials published in Chinese. Second, the meta-analysis by Zhao et al. included the specific trial by Deemer et al. (23), in which the OT intervention group, unfortunately, had significantly higher baseline age and APACHE-II severity scores compared to the control group—both of which are established, independent risk factors for the development of delirium in the ICU (24). This critical baseline imbalance likely introduced significant confounding, potentially masking any true beneficial effect of the OT intervention. Third, the high statistical heterogeneity reported in the Zhao et al. meta-analysis, coupled with their reported instability in sensitivity analyses, suggests that their pooled null result may have been less reliable. The proposed biological and psychological mechanisms through which OT may effectively prevent delirium are conceptually sound and plausible; targeted cognitive stimulation, systematic reorientation techniques, and the promotion of a normalized sleep–wake cycle through the provision of structured, meaningful daytime activity can directly counteract several key, modifiable risk factors for delirium in the vulnerable ICU population (25, 43, 44). Our current findings, derived from a set of studies exhibiting low statistical heterogeneity, substantially strengthen the argument for a dedicated role of OT in multimodal delirium prevention strategies.

The observed reduction in the duration of mechanical ventilation associated with OT is a finding of considerable clinical and economic importance. Prolonged mechanical ventilation is independently associated with deleterious sequelae including diaphragm dysfunction (specifically ventilator-induced diaphragmatic dysfunction, VIDD) (26), an elevated risk of extubation failure, and protracted ICU and hospital lengths of stay (27, 28). While the pooled effect was reported as an SMD of −0.68, which represents a moderate-to-large effect size statistically, its clinical interpretation is paramount. This SMD suggests that OT intervention shifts the distribution of ventilation duration meaningfully downwards. In practical terms, for a typical ICU patient, this could translate to a reduction of several hours to a day or more off the ventilator, a change that is clinically significant given the risks associated with each additional day of mechanical ventilation. By safely facilitating early mobilization and actively engaging accessory respiratory muscles through upright activities, postural changes, and task-oriented ADL tasks, OT may help preserve diaphragmatic contractile function and improve overall respiratory drive and coordination, thereby potentially reducing ventilator dependence and facilitating faster, more successful weaning (29, 44, 45). This functional, activity-based approach appears to complement the more passive range-of-motion exercises that are often the primary focus of standard early mobility protocols, potentially offering a valuable synergistic effect on respiratory recovery.

Furthermore, the statistically significant and clinically relevant improvement in grip strength, a reliable, objective indicator of overall limb muscle strength and a widely used proxy measure for the severity of ICU-acquired weakness (30, 31), strongly underscores the tangible role of OT in addressing the profound physical sequelae of critical illness. The task-oriented, functionally relevant exercises that are inherent to OT, such as repetitively grasping and manipulating objects of varying properties (32), provide a form of targeted, functional resistance training that can effectively stimulate the rebuilding of muscle mass, improve neuromuscular control, and enhance fine motor coordination.

### 4.1 Limitations

The conclusions of this review should be interpreted in light of its limitations, which can be categorized into those inherent to the primary included studies and those specific to this systematic review itself.

#### 4.1.1 Limitations of the primary studies

First, the inherent nature of complex behavioral interventions like OT makes blinding of participants and therapists pragmatically impossible, leading to a universally high risk of performance bias across all included trials. This unavoidable limitation could potentially inflate the perceived effect sizes due to placebo effects, heightened attention in the intervention group, or differential care provided by unblinded staff (33, 34). Second, the modest number of RCTs and the consequent total sample size for some outcomes (e.g., *n* = 3 for mechanical ventilation duration) limit the precision of the pooled estimates, as reflected in the wider confidence intervals, and reduce the generalizability of the findings. This imprecision is a key factor moderating the certainty of the evidence. Furthermore, while low heterogeneity was observed for most outcomes, a degree of clinical heterogeneity undoubtedly existed across the studies concerning the specific components (35), intensity, and duration of the OT protocols, as well as the underlying patient populations and ICU settings, which could not be fully accounted for in the analysis.

#### 4.1.2 Limitations of the systematic review

First, despite our comprehensive and systematic search across multiple international and Chinese databases, the small number of studies (<10) available for each outcome meta-analysis precluded a reliable assessment of publication bias using funnel plots or statistical tests (e.g., Egger’s test) (36). Consequently, the potential for an overestimation of effects due to unpublished negative or null findings remains unknown and constitutes a limitation of this synthesis. Second, we observed substantial statistical heterogeneity (I^2^ = 56%) for the ADL outcome. While we conducted pre-specified subgroup analyses which identified the type of assessment tool (Barthel Index vs. FIM) as a potential source, a substantial degree of heterogeneity remained unexplained. A more definitive exploration of heterogeneity through meta-regression was not feasible due to the limited number of studies and inconsistent reporting of potential effect modifiers (e.g., intervention intensity/duration, baseline illness severity scores like APACHE II) across the included trials (37). This residual heterogeneity tempers the interpretability and generalizability of the pooled ADL estimate. Finally, the overall certainty of the evidence, as formally assessed using the GRADE framework, was not high, ranging from low (for ADL) to moderate (for delirium, grip strength, and mechanical ventilation duration) (38). This grading was primarily driven by the serious risk of bias (performance bias) inherent in all studies and, in the case of ADL, by serious inconsistency (unexplained heterogeneity). The inherently subjective nature of patient-reported ADL performance, despite our use of SMD for pooling, also constitutes a methodological consideration.

### 4.2 Implications for practice and research

Notwithstanding these acknowledged limitations, the cumulative evidence synthesized in this review suggests that the timely integration of occupational therapy into the standard, multidisciplinary care of critically ill patients represents a promising and viable strategy for improving multidimensional recovery and potentially mitigating the burden of PICS. Based on the current evidence, we recommend that occupational therapy be considered as a core component of early rehabilitation bundles in the ICU, particularly for high-risk populations such as stroke survivors, older patients, and those requiring mechanical ventilation. Interventions should include protocolized, task-oriented ADL training and cognitive stimulation, initiated as early as clinically feasible (39). For current clinical practice, our findings provide a compelling evidence base to support the active consideration and implementation of early, protocolized OT interventions as an integral component of bundled care strategies aimed at preventing and managing the complex sequelae of critical illness. Occupational therapists, with their unique expertise in functional recovery, cognitive rehabilitation, and psychosocial support, should be considered integral members of the modern ICU multidisciplinary team. Future research in this field should unequivocally focus on conducting larger, rigorously designed, multicenter RCTs that employ standardized, yet potentially adaptable, OT intervention protocols to confirm and extend these promising findings. These future trials should prioritize the implementation of robust blinding procedures for outcome assessors and should place a strong emphasis on measuring patient-centered outcomes that matter most to survivors, such as long-term quality of life, successful return to work, and community reintegration (40). It would be particularly valuable to investigate the optimal timing of OT initiation (e.g., very early vs. early), the ideal daily frequency and total duration of sessions, and its differential impact on specific, well-defined patient subgroups (e.g., surgical versus medical ICU patients, patients with versus without pre-existing baseline cognitive impairment). Exploring the long-term sustainability of the benefits conferred by ICU-based OT interventions, particularly their effect on functional status and quality of life at 6 and 12 months post-discharge, represents another critical and clinically relevant avenue for future investigation. Additionally, well-conducted economic evaluations and formal cost-effectiveness analyses are urgently warranted to determine the economic value and potential resource implications of implementing dedicated ICU OT programs within different healthcare systems (41). Finally, while the current review demonstrates the clinical benefits of OT, the economic implications of implementing such services in the ICU remain largely unexplored. Future research should include formal cost-effectiveness analyses to evaluate the long-term economic value of OT interventions, considering potential reductions in ventilator days, ICU length of stay, and the burden of long-term disability (42), which would provide crucial information for healthcare policymakers and administrators.

## 5 Conclusion

In summary, this systematic review and meta-analysis provides a synthesized evaluation of the current evidence, indicating that occupational therapy significantly improves recovery in critically ill ICU patients by enhancing functional independence (with low certainty evidence), reducing delirium (moderate certainty), increasing muscle strength (moderate certainty), and shortening mechanical ventilation duration (moderate certainty). Subgroup analyses indicate stroke patients may benefit most, highlighting OT’s particular value in neurocritical care. These findings affirm OT’s important role in addressing post-ICU syndrome within interdisciplinary teams. Despite methodological limitations, the consistent benefits provide a strong case for integrating OT into standard critical care rehabilitation, though the low to moderate certainty of evidence underscores the need for further rigorous, large-scale trials to establish optimal protocols and strengthen the evidence base.

## Data Availability

The original contributions presented in the study are included in the article/Supplementary material, further inquiries can be directed to the corresponding author/s.
