# Supplementary material for: Occupational therapy improves functional recovery and reduces delirium in critically ill adults with and without stroke: a systematic review and meta-analysis
Source: Front Med (Lausanne). 2026 Feb 19;12:1733103. doi: 10.3389/fmed.2025.1733103 (PMC12961616; doi:10.3389/fmed.2025.1733103)
Supplement: Supplementary file 1 [file Table_1.DOCX]

| **Supplementary Table 1. Search Strategy** | |
| --- | --- |
| **Database** | **Search Strategy** |
| PubMed | (("Randomized Controlled Trial"[Publication Type] OR "Randomized Controlled Trials as Topic"[Mesh] OR ("random*"[Title/Abstract] OR "controlled clinical trial"[Title/Abstract] OR RCT[Title/Abstract])) AND (("Occupational Therapy"[Mesh] OR ("Occupational Therap*"[Title/Abstract] OR "occupational training"[Title/Abstract] OR "Ergotherap*"[Title/Abstract] OR "activit* of daily living"[Title/Abstract] OR "ADL training"[Title/Abstract] OR "skilled treatment"[Title/Abstract] OR "independent living"[Title/Abstract] OR "occupation based"[Title/Abstract] OR "occupation centered"[Title/Abstract] OR "activity focused"[Title/Abstract] OR "purposeful activity"[Title/Abstract] OR "vocational rehabilitation"[Title/Abstract])) AND (((("Intensive Care Units"[Mesh] OR "Critical Care"[Mesh] OR "Critical Illness"[Mesh]) OR ("Intensive Care"[Title/Abstract] OR ICU[Title/Abstract] OR "critical*ill*"[Title/Abstract] OR "critical care"[Title/Abstract])))) |
| Embase | #1 'intensive care unit'/exp #2 'intensive care'/exp #3 'critical illness'/exp #4 'intensive care':ti,ab,kw OR icu:ti,ab,kw OR 'critical*ill*':ti,ab,kw OR 'critical care':ti,ab,kw #5 #1 OR #2 OR #3 OR #4 #6 'occupational therapy'/exp #7 'occupational therap*':ti,ab,kw OR 'occupational training':ti,ab,kw OR 'ergotherap*':ti,ab,kw OR 'activit* of daily living':ti,ab,kw OR 'adl training':ti,ab,kw OR 'skilled treatment':ti,ab,kw OR 'independent living':ti,ab,kw OR 'occupation based':ti,ab,kw OR 'occupation centered':ti,ab,kw OR 'activity focused':ti,ab,kw OR 'purposeful activity':ti,ab,kw OR 'vocational rehabilitation':ti,ab,kw #8 #6 OR #7 #9 'randomized controlled trial'/exp #10 'random*':ti,ab,kw OR 'controlled clinical trial':ti,ab,kw OR rct:ti,ab,kw #11 #9 OR #10 #12 #5 AND #8 AND #11 |
| Cochrane | #1 MeSH descriptor: [Intensive Care Units] explode all trees #2 MeSH descriptor: [Critical Care] explode all trees #3 MeSH descriptor: [Critical Illness] explode all trees #4 (Intensive Care):ti,ab,kw OR (ICU):ti,ab,kw OR (critical*il*):ti,ab,kw OR (critical care):ti,ab,kw #5 #1 OR #2 OR #3 OR #4 #6 MeSH descriptor: [Occupational Therapy] explode all trees #7 (Occupational Therap*):ti,ab,kw OR (occupational training):ti,ab,kw OR (Ergotherap*):ti,ab,kw OR (activit* of daily living):ti,ab,kw OR (ADL training):ti,ab,kw #8 (skilled treatment):ti,ab,kw OR (independent living):ti,ab,kw OR (occupation based):ti,ab,kw OR (occupation centered):ti,ab,kw OR (activity focused):ti,ab,kw #9 (purposeful activity):ti,ab,kw OR (vocational rehabilitation):ti,ab,kw #10 #6 OR #7 OR #8 OR #9 #11 MeSH descriptor: [Randomized Controlled Trial] explode all trees #12 (random*):ti,ab,kw OR (controlled clinical trial):ti,ab,kw OR (RCT):ti,ab,kw #13 #11 OR #12 #14 #5 AND #10 AND #13 |
| Web of Science | #1 TS=("Intensive Care" OR ICU OR "critical*il*" OR "critical care") #2 TS=("Occupational Therap*" OR "occupational training" OR "Ergotherap*" OR "activit* of daily living" OR "ADL training" OR "skilled treatment" OR "independent living" OR "occupation based" OR "occupation centered" OR "activity focused" OR "purposeful activity" OR "vocational rehabilitation") #3 TS=("random*" OR "controlled clinical trial" OR RCT) #4 #3 AND #2 AND #1 |
| CINAHL | S1 MH ((MH "Intensive Care Units") OR (MH "Critical Illness") OR (MH "Critical Care")) OR AB ("Intensive Care" OR ICU OR "critical*ill*" OR "critical care") OR TI ("Intensive Care" OR ICU OR "critical*ill*" OR "critical care") S2 MH "Occupational Therapy" OR TI (Occupational Therap* OR "occupational training" OR "Ergotherap*" OR "activit* of daily living" OR "ADL training" OR "skilled treatment" OR "independent living" OR "occupation based" OR "occupation centered" OR "activity focused" OR "purposeful activity" OR "vocational rehabilitation") OR AB (Occupational Therap* OR "occupational training" OR "Ergotherap*" OR "activit* of daily living" OR "ADL training" OR "skilled treatment" OR "independent living" OR "occupation based" OR "occupation centered" OR "activity focused" OR "purposeful activity" OR "vocational rehabilitation") S3 MH "Randomized Controlled Trials" OR TI ("random*" OR "controlled clinical trial" OR RCT) OR AB ("random*" OR "controlled clinical trial" OR RCT) S4 S1 AND S2 AND S3 |
| SinoMed | "随机"[常用字段] AND ("作业治疗"[常用字段] OR "作业训练"[常用字段] OR "职业治疗"[常用字段] OR "职业训练"[常用字段] OR ("日常生活活动"[常用字段] OR "Activities of Daily Living"[常用字段] OR "ADL"[常用字段] OR "长期活动限度"[常用字段] OR "慢性活动的限制"[常用字段] OR "日常生活活动"[主题词]) OR "ADL 训练"[常用字段] OR "技能训练"[常用字段] OR ("独立生活"[常用字段] OR "Independent Living"[常用字段] OR "聚居"[常用字段] OR "原居安老"[常用字段] OR "独立生活"[主题词]) OR ("职业康复"[常用字段] OR "职业性康复"[常用字段] OR "Vocational Rehabilitation"[常用字段] OR "康复, 职业性"[主题词])) AND ("ICU"[常用字段] OR "重症监护室"[常用字段] OR "监护病房"[常用字段] OR "重症"[常用字段] OR "危重"[常用字段]) |
| Wanfang Database | 主题: (ICU OR 重症监护室 OR 监护病房 OR 重症 OR 危重) AND 主题: (作业治疗 OR 作业训练 OR 职业治疗 OR 职业训练 OR 日常生活活动 OR ADL 训练 OR 技能训练 OR 独立生活 OR 职业康复) AND 主题: (随机) |
| CNKI | (篇关摘: ICU + 重症监护室 + 监护病房 + 重症 + 危重 (模糊)) AND (篇关摘: 作业治疗 + 作业训练 + 职业治疗 + 职业训练 + 日常生活活动 + ADL 训练 + 技能训练 + 独立生活 + 职业康复 (模糊)) AND (篇关摘: 随机 (模糊)) |
