# Supplementary material for: Occupational therapy improves functional recovery and reduces delirium in critically ill adults with and without stroke: a systematic review and meta-analysis
Source: Front Med (Lausanne). 2026 Feb 19;12:1733103. doi: 10.3389/fmed.2025.1733103 (PMC12961616; doi:10.3389/fmed.2025.1733103)
Supplement: Supplementary file 2 [file Table_2.DOCX]

**Supplementary Table 2. Subgroup Analysis: Summary of Meta-Analysis Results for Patients with Stroke**

**Supplementary Table 2a. Effect of Occupational Therapy on Activities of Daily Living (ADL) in Stroke Patients**

| Study or Subgroup | Experimental Group | Control Group | Std. Mean Difference | Std. Mean Difference |  |  |  |  |
| --- | --- | --- | --- | --- | --- | --- | --- | --- |
|  | Mean | SD | Total | Mean | SD | Total | SMD | 95% CI |
| Jiang 2018 | 89.95 | 17.42 | 46 | 74.02 | 16.39 | 43 | 0.93 | [0.49, 1.37] |
| Ping Yu 2018 | 48.29 | 9.95 | 38 | 38.09 | 6.86 | 34 | 1.17 | [0.67, 1.67] |
| Dan Tu 2020 | - | - | 33 | - | - | 33 | 0.49 | [0.00, 0.98] |
| Total (95% CI) | 117 |  | 110 |  |  |  | 0.81 | [0.42, 1.20] |

**Heterogeneity:** Chi² = 2.85, df = 2 (P = 0.24); I² = 30%
**Test for overall effect:** Z = 4.10 (P < 0.001)

**Supplementary Table 2b. Effect of Occupational Therapy on Incidence of Delirium in Stroke Patients**

| Study or Subgroup | Experimental Group | Control Group | Risk Ratio | Risk Ratio |  |  |
| --- | --- | --- | --- | --- | --- | --- |
|  | Events | Total | Events | Total | RR | 95% CI |
| Jiang 2018 | 4 | 46 | 9 | 43 | 0.41 | [0.14, 1.26] |
| Ping Yu 2018 | 8 | 38 | 15 | 34 | 0.48 | [0.23, 0.98] |
| Dan Tu 2020 | 10 | 33 | 18 | 33 | 0.56 | [0.30, 1.02] |
| Total (95% CI) | 117 |  | 110 |  | 0.39 | [0.21, 0.72] |
| Total events | 22 |  | 42 |  |  |  |

**Heterogeneity:** Chi² = 0.33, df = 2 (P = 0.85); I² = 0%
**Test for overall effect:** Z = 3.00 (P = 0.003)

**Notes for Supplementary Table X:**

- **Population Definition:** The "Stroke" subgroup was defined as patients admitted to the ICU primarily for acute stroke or with a primary neurosurgical diagnosis (e.g., cerebral hemorrhage, ischemic stroke). This subgroup was pre-specified in the analysis protocol.
- **Data Imputation:** For the study by Dan Tu 2020 in Table Xa, the exact mean and SD for the ADL outcome were not reported in the original publication. The effect size (SMD) was estimated based on the reported P-value and group sample sizes to allow for inclusion in the quantitative synthesis. Corresponding authors were contacted for original data but no response was received.
- **Statistical Model:** A **random-effects** model was used for the ADL meta-analysis due to moderate heterogeneity, and a **fixed-effect** model was used for the delirium meta-analysis due to negligible heterogeneity.
- **Interpretation:** The pooled results indicate that occupational therapy has a **large, significant effect** on improving ADL (SMD > 0.8) and is associated with a **61% reduction** in the relative risk of delirium in critically ill patients with stroke.
