# Supplementary material for: Occupational therapy improves functional recovery and reduces delirium in critically ill adults with and without stroke: a systematic review and meta-analysis
Source: Front Med (Lausanne). 2026 Feb 19;12:1733103. doi: 10.3389/fmed.2025.1733103 (PMC12961616; doi:10.3389/fmed.2025.1733103)
Supplement: Supplementary file 3 [file Table_3.DOCX]

| **Supplementary Table 3. GRADE Evidence Profile: Occupational Therapy for Critically Ill Adults in the ICU** | | | | |  |
| --- | --- | --- | --- | --- | --- |
| **Outcomes** | Anticipated Absolute effects (95% CI)* | **№ of participants (studies)** | **Certainty of the evidence (GRADE)** | **Comments** |  |
|  | **Risk with Control** | **Risk with Occupational Therapy** |  |  |  |
| **Activities of Daily Living (ADL)** | - | **SMD 0.72 higher (0.40 higher to 1.05 higher)** | 757 (5 RCTs) | **@@○○ LOW^^^a,b** | **OT likely improves ADL performance, but the true effect may be substantially different.** |
| **Incidence of Delirium** | 333 per 1,000 | 146 per 1,000 (100 to 210) | 698 (4 RCTs) | **@@@○ MODERATE^^^a** | **OT likely reduces the incidence of delirium.** |
|  |  | **RR 0.44 (0.30 to 0.63)** |  |  |  |
| **Grip Strength (kg)** | - | **MD 3.90 kg higher (2.03 higher to 5.76 higher)** | 400 (4 RCTs) | **@@@○ MODERATE^^^a** | **OT likely improves grip strength.** |
| **Duration of Mechanical Ventilation** | - | **SMD 0.68 lower (0.99 lower to 0.37 lower)** | 461 (3 RCTs) | **@@@○ MODERATE^^^a** | **OT likely reduces the duration of mechanical ventilation.** |

**Explanation:**

- **CI:** Confidence interval; **RR:** Risk ratio; **SMD:** Standardised mean difference; **MD:** Mean difference
- **GRADE Working Group grades of evidence**
  - **High certainty (@@@@):** We are very confident that the true effect lies close to that of the estimate of the effect.
  - **Moderate certainty (@@@○):** We are moderately confident in the effect estimate: The true effect is likely to be close to the estimate of the effect, but there is a possibility that it is substantially different.
  - **Low certainty (@@○○):** Our confidence in the effect estimate is limited: The true effect may be substantially different from the estimate of the effect.
  - **Very low certainty (@○○○):** We have very little confidence in the effect estimate: The true effect is likely to be substantially different from the estimate of effect.

**Reasons for downgrading:**
^a: **Downgraded for risk of bias:** All studies were judged at high risk of performance bias due to the inability to blind participants and personnel. For objectively measured outcomes (grip strength, ventilation duration) where assessor blinding was often reported, this was the primary limitation. For more subjective outcomes (ADL), the risk of bias was considered higher, but the primary reason for downgrading ADL further was inconsistency.
^b: **Downgraded for inconsistency:** Substantial statistical heterogeneity was observed (I² = 56%) which was not fully explained by pre-specified subgroup analyses.
